# Impacts of Swachh Bharat Mission on Health and Communicable Disease Control: A Systematic Review Protocol

**DOI:** 10.1002/hsr2.70425

**Published:** 2025-02-06

**Authors:** Karan Varshney, Chirag Khatri, Prabhat Lamichhane

**Affiliations:** ^1^ School of Medicine Deakin University Geelong Australia; ^2^ School of Public Health and Preventive Medicine Monash University Melbourne Australia; ^3^ Department of Public Health La Trobe University Melbourne Australia

**Keywords:** communicable disease, infection, sanitation, Swachh Bharat

## Abstract

**Background and Aims:**

Swachh Bharat Mission (SBM) is a major initiative led by the Government of Bharat (India) in 2014 to improve sanitation across the country. While the government has received international praise for SBM's achievements, the health impacts of this initiative are not well understood, and to date, no systematic review has been conducted on the topic. In this protocol, we aim to describe our methodology for systematically reviewing the literature to determine the impacts of SBM on communicable disease control and population health outcomes across Bharat.

**Methods:**

This protocol describes the process that will be taken for our systematic review. The review will follow the Preferred Reporting Items for Systematic review and Meta‐Analysis guidelines. Searches will be conducted in Scopus, Embase, Medline, Web of Science, Cumulated Index to Nursing and Allied Health Literature, PubMed, and Global Health. Original, full‐text research articles conducted after October 2014 (the enactment of SBM) will be eligible for inclusion, as will be gray literature. Modeling studies will be excluded from this review. Studies which analyze impacts/trends relating to health or communicable disease control in any region of Bharat will be eligible for inclusion Included studies will assess at least one of the following: impact on infectious disease incidence/prevalence and morbidity/mortality, infant and under‐five mortality, nutritional impacts (including stunting, underweight and wasting) or non‐communicable disease rates. Study quality will be assessed using the Kmet checklist. Where possible, data will be pooled and synthesized to determine overall findings and trends.

**Discussion:**

This review will highlight the extent to which SBM has, or has not, had lasting impacts on health and communicable disease control. The findings of this review can have important implications in shaping and guiding the ongoing implementation of SBM across Bharat.

## 1 Background

Nearly 500,000 deaths are estimated to occur annually due to contaminated sources of drinking water in the worldwide [1, 2]. These death totals are attributable to preventable water related illnesses like cholera, dysentery, and typhoid [1] but these numbers do not account for additional morbidity and mortality from respiratory infections, soil‐transmitted helminths (STHs) and pollutants, which have been estimated to increase the total death toll to more than 800,000 deaths [3]. Factors contributing to mortality for these populations who deal with these health issues are a lack of toilets, poor housing, and a lack of adequate plumbing [3]. Improving of water pollution may have a role in reducing the burden of these infections overall [3].

There are also notable financial costs across society that are associated with water contamination and poor overall water quality. It has been estimated that the annual, worldwide economic costs of poor sanitation are approximately more than US $142 billion [4] For example, In Cambodia, losses of approximately US $4.8 million per year have occurred due to poor sanitation [4]; In Indonesia, the estimated annual costs are US $3.3 billion per year [4]. Furthermore, it has been demonstrated that water contamination and pollution can result in up to a 2% decrease in economic growth of nations [5].

A major cause of water contamination and pollution is the practice of open defecation by humans, defined as when human feces are disposed of in fields, forests, bushes, open bodies of water, beaches or other open spaces or disposed of with solid waste [6]. Other important contributors to water contamination include other poor waste disposal and inadequate sewage treatment, agricultural products/waste (including animal fecal matter), runoff from paved surfaces and roads, and dumping of organic pollutants into water reservoirs [7].

A lack of sanitation infrastructure to ensure access to usable, clean water remains an issue across the world. Low‐ and Middle‐Income countries continue to face these challenges despites decades of investment and effort [2, 6]. Limited access to adequate sanitation in Bharat (India) has also been well documented [8, 9, 10]. For example, the 2011 Census of India demonstrated that some states' populations have latrine coverage that is only 22% [10]. There have been numerous attempts to address the challenge in previous decades, with little success. For example, initiatives such as the 1986 Central Rural Sanitation Program, the 1999 Total Sanitation Campaign, and the Nirmal Bharat Abhiyan have been proven to be unsuccessful [11]. Reasons attributed to the lack of success for these initiatives were limited allocation of funds for geographic areas of need, misuse of funds that should have been used for developing latrines, and a lack of motivation to ensure ongoing chance after initial successes [11].

On October 2, 2014, the national government of Bharat enacted a large‐scale initiative, Swachh Bharat Mission (SBM) or Swachh Bharat Abhiyan to “Clean India” [12]. This initiative aims to bring about an improvement in the general quality of life in the rural areas, by promoting cleanliness, hygiene and eliminating open defecation. Currently, there are government financial incentives for communities to acquire solid and liquid waste management, methods to incorporate community members in roles to promote and sustain hygiene practices and long‐term goals to imbed hygiene into Indian culture [13, 14]; the aims and goals of SBM have aligned with UNICEF's emphasis of the importance of clean water and sanitation for the entirety of the populace [12]. Thus far, during the period of 2014‐15 to 2019‐20, the total investment made for these efforts was approximately 937 billion INR [13, 14].

To date, the enactment of SBM by Narendra Modi's government has been praised for its potential to greatly improve population health outcomes across Bharat [15, 16, 17]. However, SBM has been subject to various critiques, with some stating that attitudes and behaviors have not been impacted enough, and that access to sanitation and clean water remains limited for numerous segments of the population [18, 19, 20, 21]. Furthermore, while modeling studies have predicted potential health impacts of SBM since its implementation in October 2014 [16, 17], the extent of SBM's actual lasting, and tangible, impacts on health and communicable disease remains unclear; specifically, there has been a lack of synthesis of existing studies that have evaluated SBM's impacts on health. No systematic review has been conducted on this topic thus far. The analysis of the existing evidence‐base will be critical in guiding the ongoing implementation of SBM to improve the health of populations most effectively across Bharat. Therefore, we aim to systematically review and synthesize data from studies that have assessed the impact of SBM on health outcomes and overall communicable disease control.

## 2 Methods and Analysis

This systematic review will follow the Preferred Reporting Items for Systematic review and Meta‐Analysis (PRISMA) guidelines [22], and this protocol will follow the Preferred Reporting Items for Systematic review and Meta‐Analysis Protocol (PRISMA‐P) checklist [23].

The protocol for this review has been registered in the Open Science framework and is available at: https://osf.io/nw4uv. As this is research that does not involve human participants, ethical approval and informed consent is not required for this work.

### 2.1 Database Searches

Searches will be conducted in a total of seven different databases: Scopus, Embase, Medline, Web of Science, Cumulated Index to Nursing and Allied Health Literature (CINAHL), PubMed, and Global Health. Additional potentially relevant articles identified in reference lists of articles retrieved will also be considered, and gray literature sources will also be retrieved.

Search terms will utilize variations of the term “Swachh Bharat Mission,” and will include terms focusing on communicable disease, infection, and impacts on health. MeSH terms (or their equivalent per respective database), along with the Boolean operators “OR” and “AND” will be used for searches. Table 1 shows the list of search strings that will be used, by database.

**Table 1:** Search string by respective database.

Database	Search terms
**Scopus**	(TITLE‐ABS‐KEY ({Swachh Bharat Mission}) OR TITLE‐ABS‐KEY ({Swachh Bharat}) OR TITLE‐ABS‐KEY ({Swachh Bharat Abhiyan}) OR TITLE‐ABS‐KEY ({Clean India Mission}) OR TITLE‐ABS‐KEY ({Campaign Clean India}) OR TITLE‐ABS‐KEY ({Swachh Bharat Yojana}) OR TITLE‐ABS‐KEY ({Clean India}) OR TITLE‐ABS‐KEY ({Open Defecation Free}) OR TITLE‐ABS‐KEY ({sanitation campaign})) AND (TITLE‐ABS‐KEY ({Infections}) OR TITLE‐ABS‐KEY ({Coinfection}) OR TITLE‐ABS‐KEY ({Infectious Disease Transmission}) OR TITLE‐ABS‐KEY ({Infectious Disease}) OR TITLE‐ABS‐KEY ({Waterborne Diseases}) OR TITLE‐ABS‐KEY ({Communicable Diseases}) OR TITLE‐ABS‐KEY ({Bacteria}) OR TITLE‐ABS‐KEY ({Viruses}) OR TITLE‐ABS‐KEY ({Pathogens}) OR TITLE‐ABS‐KEY ({Diarrhea}) OR TITLE‐ABS‐KEY ({Microbes}) OR TITLE‐ABS‐KEY ({Prevalence}) OR TITLE‐ABS‐KEY ({Epidemiology}) OR TITLE‐ABS‐KEY ({Incidence}) OR TITLE‐ABS‐KEY ({Health Impact Assessment}) OR TITLE‐ABS‐KEY ({Demography}) OR TITLE‐ABS‐KEY ({Mortality}) OR TITLE‐ABS‐KEY ({Health Status}) OR TITLE‐ABS‐KEY ({Health Status Indicators}) OR TITLE‐ABS‐KEY ({Death}) OR TITLE‐ABS‐KEY ({Infant Death}) OR TITLE‐ABS‐KEY ({Recurrence}) OR TITLE‐ABS‐KEY ({Population Health}) OR TITLE‐ABS‐KEY ({Life Expectancy}) OR TITLE‐ABS‐KEY ({Disability‐Adjusted Life Years}) OR TITLE‐ABS‐KEY ({Critical Care Outcomes}) OR TITLE‐ABS‐KEY ({Treatment Outcomes}) OR TITLE‐ABS‐KEY ({Fatal Outcome}) OR TITLE‐ABS‐KEY ({Disease Outbreaks}) OR TITLE‐ABS‐KEY ({Epidemics}) OR TITLE‐ABS‐KEY ({Health Impacts}) OR TITLE‐ABS‐KEY ({Health Outcomes}))
**Embase**	**Search #1:** ‘Swachh Bharat Mission’/exp OR ‘Swachh Bharat’/exp OR ‘Swachh Bharat Abhiyan’/exp OR ‘Clean India Mission’/exp OR ‘Campaign Clean India’/exp OR ‘Swachh Bharat Yojana’/exp OR ‘Clean India’/exp OR ‘Open Defecation Free’/exp OR ‘sanitation campaign’/exp *AND* **Search #2:** ‘infections’/exp OR ‘coinfection’/exp OR ‘disease transmission, infectious’/exp OR ‘infectious disease transmission, vertical’/exp OR ‘infectious diseases’/exp OR ‘waterborne diseases’/exp OR ‘communicable diseases’/exp OR ‘bacteria’/exp OR ‘viruses’/exp OR ‘pathogens’/exp OR ‘diarrhea’/exp OR ‘microbes’/exp OR ‘prevalence’/exp OR ‘epidemiology’/exp OR ‘incidence’/exp OR ‘health impact assessment’/exp OR ‘demography’/exp OR ‘mortality’/exp OR ‘health status’/exp OR ‘health status indicators’/exp OR ‘death’/exp OR ‘infant death’/exp OR ‘recurrence’/exp OR ‘population health’/exp OR ‘life expectancy’/exp OR ‘disability‐adjusted life years’/exp OR ‘critical care outcomes’/exp OR ‘treatment outcome’/exp OR ‘fatal outcome’/exp OR ‘disease outbreaks’/exp OR ‘epidemics’/exp OR ‘health impacts’ OR ‘health outcomes’/exp
**Cumulated Index to Nursing and Allied Health Literature (CINAHL)**	(TX(Swachh Bharat Mission* OR Swachh Bharat* OR Swachh Bharat Abhiyan* OR Clean India Mission* OR Campaign Clean India* OR Swachh Bharat Yojana* OR Clean India* OR Open Defecation Free* OR sanitation campaign*)) AND (Infections.mp,hw. OR Coinfection.mp,hw. OR Disease Transmission, Infectious.mp,hw. OR Infectious Disease Transmission, Vertical.mp,hw. OR Infectious Diseases.mp,hw. OR Waterborne Diseases.mp,hw. OR Communicable Diseases.mp,hw. OR Bacteria.mp,hw. OR Viruses.mp,hw. OR Pathogens.mp,hw. OR Diarrhea.mp,hw. OR Microbes.mp,hw. OR Prevalence.mp,hw. OR Epidemiology.mp,hw. OR Incidence.mp,hw. OR Health Impact Assessment.mp,hw. OR Demography.mp,hw. OR Mortality.mp,hw. OR Health Status.mp,hw. OR Health Status Indicators.mp,hw. OR Death OR Infant Death.mp,hw. OR Recurrence.mp,hw. OR Population Health.mp,hw. OR Life Expectancy.mp,hw. OR Disability‐Adjusted Life Years.mp,hw. OR Critical Care Outcomes.mp,hw. OR Treatment Outcome.mp,hw. OR Fatal Outcome.mp,hw. OR Disease Outbreaks.mp,hw. OR Epidemics.mp,hw. OR Health Impacts.mp,hw. OR Health Outcomes.mp,hw.)
**Medline**	(TX(Swachh Bharat Mission* OR Swachh Bharat* OR Swachh Bharat Abhiyan* OR Clean India Mission* OR Campaign Clean India* OR Swachh Bharat Yojana* OR Clean India* OR Open Defecation Free* OR sanitation campaign*)) AND (Infections.mp,hw. OR Coinfection.mp,hw. OR Disease Transmission, Infectious.mp,hw. OR Infectious Disease Transmission, Vertical.mp,hw. OR Infectious Diseases.mp,hw. OR Waterborne Diseases.mp,hw. OR Communicable Diseases.mp,hw. OR Bacteria.mp,hw. OR Viruses.mp,hw. OR Pathogens. mp, hw. OR Diarrhea.mp,hw. OR Microbes.mp,hw. OR Prevalence.mp,hw. OR Epidemiology.mp,hw. OR Incidence.mp,hw. OR Health Impact Assessment.mp,hw. OR Demography.mp,hw. OR Mortality.mp,hw. OR Health Status.mp,hw. OR Health Status Indicators.mp,hw. OR Death OR Infant Death.mp,hw. OR Recurrence.mp,hw. OR Population Health.mp,hw. OR Life Expectancy.mp,hw. OR Disability‐Adjusted Life Years.mp,hw. OR Critical Care Outcomes.mp,hw. OR Treatment Outcome.mp,hw. OR Fatal Outcome.mp,hw. OR Disease Outbreaks.mp,hw. OR Epidemics.mp,hw. OR Health Impacts.mp,hw. OR Health Outcomes. mp, hw.)
**Web of Science**	(“Swachh Bharat Mission” OR “Swachh Bharat” OR “Swachh Bharat Abhiyan” OR “Clean India Mission” OR “Campaign Clean India” OR “Swachh Bharat Yojana” OR “Clean India” OR “Open Defecation Free” OR “sanitation campaign”) AND (“Infections” OR “Coinfection” OR “Disease Transmission, Infectious” OR “Infectious Disease Transmission, Vertical” OR “Infectious Diseases” OR “Waterborne Diseases” OR “Communicable Diseases” OR “Bacteria” OR “Viruses” OR “Pathogens” OR “Diarrhea” OR “Microbes” OR “Prevalence” OR “Epidemiology” OR “Incidence” OR “Health Impact Assessment” OR “Demography” OR “Mortality” OR “Health Status” OR “Health Status Indicators” OR “Death” OR “Infant Death” OR “Recurrence” OR “Population Health” OR “Life Expectancy” OR “Disability‐Adjusted Life Years” OR “Critical Care Outcomes” OR “Treatment Outcome” OR “Fatal Outcome” OR “Disease Outbreaks” OR “Epidemics” OR “Health Impacts” OR “Health Outcomes”)
**Global Health**	(TX(Swachh Bharat Mission* OR Swachh Bharat* OR Swachh Bharat Abhiyan* OR Clean India Mission* OR Campaign Clean India* OR Swachh Bharat Yojana* OR Clean India* OR Open Defecation Free* OR sanitation campaign*)) AND (Infections.mp,hw. OR Coinfection.mp,hw. OR Disease Transmission, Infectious.mp,hw. OR Infectious Disease Transmission, Vertical.mp,hw. OR Infectious Diseases.mp,hw. OR Waterborne Diseases.mp,hw. OR Communicable Diseases.mp,hw. OR Bacteria.mp,hw. OR Viruses.mp,hw. OR Pathogens.mp,hw. OR Diarrhea.mp,hw. OR Microbes.mp,hw. OR Prevalence.mp,hw. OR Epidemiology.mp,hw. OR Incidence.mp,hw. OR Health Impact Assessment.mp,hw. OR Demography.mp,hw. OR Mortality.mp,hw. OR Health Status.mp,hw. OR Health Status Indicators.mp,hw. OR Death OR Infant Death.mp,hw. OR Recurrence.mp,hw. OR Population Health.mp,hw. OR Life Expectancy.mp,hw. OR Disability‐Adjusted Life Years.mp,hw. OR Critical Care Outcomes.mp,hw. OR Treatment Outcome.mp,hw. OR Fatal Outcome.mp,hw. OR Disease Outbreaks.mp,hw. OR Epidemics.mp,hw. OR Health Impacts.mp,hw. OR Health Outcomes.mp,hw.)
**PubMed**	(“Swachh Bharat Mission”[Title/Abstract] OR “Swachh Bharat”[Title/Abstract] OR “Swachh Bharat Abhiyan”[Title/Abstract] OR “Clean India Mission”[Title/Abstract] OR “Campaign Clean India”[Title/Abstract] OR “Swachh Bharat Yojana”[Title/Abstract] OR “Clean India”[Title/Abstract] OR “Open Defecation Free”[Title/Abstract] OR “sanitation campaign”[Title/Abstract]) AND (“Infections”[Mesh] OR “Coinfection”[Mesh] OR “Disease Transmission, Infectious”[Mesh] OR “Infectious Disease Transmission, Vertical”[Mesh] OR “Infectious Diseases”[Title/Abstract] OR “Waterborne Diseases”[Mesh] OR “Communicable Diseases”[Mesh] OR “Bacteria”[Mesh] OR “Viruses”[Mesh] OR “Pathogens”[Title/Abstract] OR “Diarrhea”[Mesh] OR “Microbes”[Title/Abstract] OR “Prevalence”[Mesh] OR “Epidemiology”[Mesh] OR “Incidence”[Mesh] OR “Health Impact Assessment”[Mesh] OR “Demography”[Mesh] OR “Mortality”[Mesh] OR “Health Status”[Mesh] OR “Health Status Indicators”[Mesh] OR “Death”[Mesh] OR “Infant Death”[Mesh] OR “Recurrence”[Mesh] OR “Population Health”[Mesh] OR “Life Expectancy”[Mesh] OR “Disability‐Adjusted Life Years”[Mesh] OR “Critical Care Outcomes”[Mesh] OR “Treatment Outcome”[Mesh] OR “Fatal Outcome”[Mesh] OR “Disease Outbreaks”[Mesh] OR “Epidemics”[Mesh] OR “Health Impacts”[Title/Abstract] OR “Health Outcomes”[Title/Abstract])

### 2.2 Inclusion and Exclusion Criteria

All type of empirical quantitative studies that includes data after commencement of the SBM program (i.e., after October 2014) will be considered for inclusion in the review. Studies that only include data before October 2014 will be excluded from this review. Studies that assess impacts on health or on communicable disease control of SBM in any region of Bharat will be eligible for inclusion. This will include studies that assess impact on infectious disease (such as helminth worms, diarrhea, and presence of water‐borne diseases), incidence/prevalence and/morbidity/mortality, infant and under‐five mortality, nutritional status (deficiencies, childhood stunting, underweight and wasting) and non‐communicable disease. Analysis of any other health related factor (such as vaccinations, mental health status) will also allow for a study to be included.

Original research studies, either in the form of primary or secondary analysis of data published in peer‐reviewed journals or available as a gray literature in English, will be considered. Review, modeling studies, and commentaries/editorials will be excluded from the review. Qualitative studies will be excluded as this review primarily aims to synthesize the quantitative impact of SBM.

### 2.3 Study Screening

**Figure 1:**
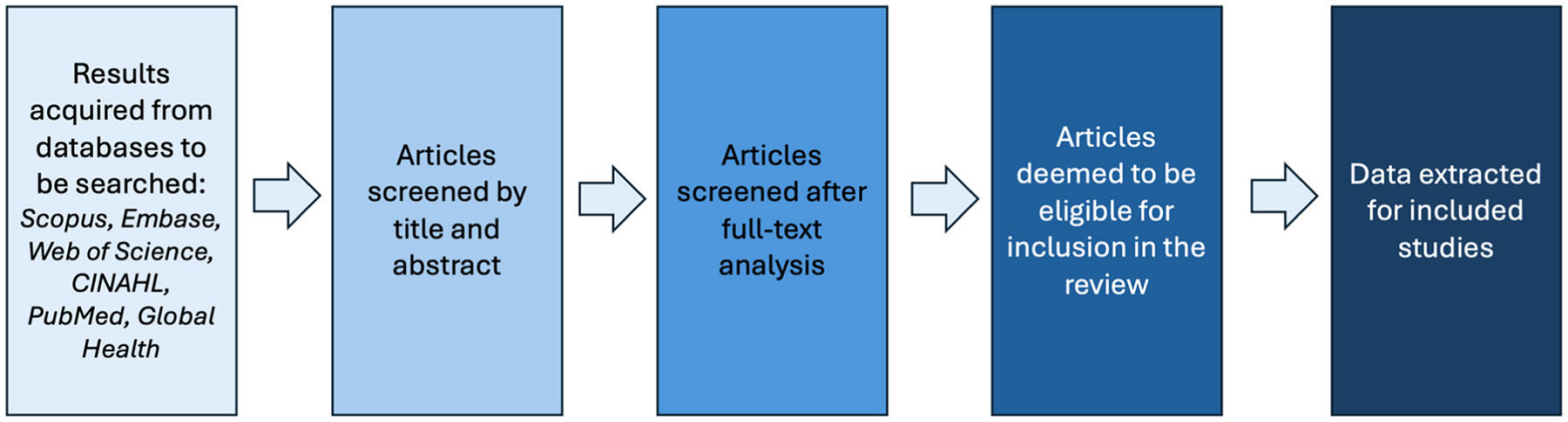
Study screening workflow based on the PRISMA guidelines [22].

The process of screening will entail first gathering articles from the combined searches. Covidence software (a collaborative software platform which streamlines the production of systematic reviews) [24] will be used for the purposes of screening. After the removal of duplicates, articles will be screened by title/abstract, and thereafter full‐text analysis. Justifications for exclusion by full text will be described in the review. Two reviewers (KV and CK) will independently screen articles; any discrepancies will be discussed and resolved by consensus in the team. A visual depiction of the study screening workflow is shown in Figure 1.

### 2.4 Data Extraction and Synthesis

Two reviewers (K.V. and C.K.) will extract the data independently once agreement is reached regarding the articles to be included in the review. Any disagreements will be resolved by consensus. Methodological study details and study findings will be extracted from the eligible studies on a data extraction form. This will include study location, objectives, study design, data sources, analytical method, reported study limitations, profile of study participants and key findings. Findings will be depicted in a tabular format and described in further detail in the text. Next, the overall results from all studies will be analyzed and described with a narrative synthesis, with trends and anomalous findings being elaborated in further detail in the review.

### 2.5 Quality Assessment

Study quality will be assessed using the Kmet checklist for quantitative studies, which is a critical appraisal tool for studies of diverse methodologies [25]. Scores will be provided based on the number of “Yes” responses (worth 2 points) or “Partial” responses (worth 1 point) on each metric for the checklist; following an approach taken previously [26], relative scores will be converted into percents and depicted graphically. Once again, two reviewers (K.V. and C.K.) will conduct this process independently and consensus will thereafter be reached.

### 2.6 Dissemination

The findings of the review will be shared and disseminated as a peer‐reviewed publication.

## 3 Discussion

There remains a clear need to understand the extent of the impacts of SBM. This review will synthesize the impact of SBM in improving population health outcomes and influencing the spread of communicable disease across Bharat and will provide important insights about the extent to which SBM has, or has not, had a role in influencing health outcomes across the country. To our knowledge, no prior review has been conducted on this topic and this will hence be the first systematic review on the topic. In this protocol, we have outlined the steps that will be taken in conducting this systematic review.

After this research has been completed, it can provide guidance regarding future research. Such research should build off the review and aim to further identify already noted health outcomes and improvements in health due to SBM. These analyzes should occur at a local level, but also at a state and national level. Future research should also focus on perceptions regarding SBM. This can allow for researchers to understand the views of SBM for people across the nation, and this can be useful in improving the initiative. Finally, there should be continued longer‐term research to evaluate all aspects of SBM. It will be useful to determine the impacts of SBM more years and decades into the future, and to use this as a guide as how water systems have improved in the past and can continue to improve in the future.

The review and its associated findings will serve as an important indicator of the success of the program and its overall shortcomings. They will also provide guidance for the SBM's past successes and can offer lessons for other comparable programs and initiatives that are taking place in Bharat. This work can hence have important impacts in impacting policy both within Bharat, and internationally in other countries that seek to improve sanitation and health outcomes relating to clean water access [27]. With the findings based on the health impacts of SBM, valuable insights may be provided for global populations regarding how waterborne disease and associated health issues can be addressed in areas where sanitation is lacking, and water contamination is substantial issue. Furthermore, as SBM is currently in its second phase, the findings from the review can have a role in guiding the ongoing implementation of SBM's second phase to ensure that that past successes in the domain of health are continued, and that past shortcomings are avoided as much as possible. Health outcomes across the nation can potentially be impacted and improved, if overall implementation and quality improvement is successful.

It is also critical to consider potential limitations that will emerge for this review. While this review will seek to include studies that analyze the impacts of SBM, it will be difficult to determine if the change in health outcomes is a result of SBM, or other events, policies, or other form of public health action. Furthermore, there may be unaddressed confounding variables, such as the lasting impacts of previous health campaigns, the co‐occurrence of other programs, or a change in gross domestic product/health spending may not be addressed in the studies included and in the review. For example, this review will likely not be able to account for the impacts of the COVID pandemic on SBM's implementation. Additionally, due to the nature of the research question and the need for quantitative health data, we will not be able to assess the lived realities for people regarding impacts of SBM via qualitative data in this review. Regardless of these limitations, this review has the potential to provide valuable insights regarding the overall health impacts of SBM. These insights can have important roles in shaping future policies in Bharat, but also in guiding the future development of policies in other contexts where a lack of adequate sanitation remains a major issue.

## Author Contributions

**Karan Varshney:** conceptualization, data curation, formal analysis, writing – original draft, methodology, writing – review and editing. **Chirag Khatri:** formal analysis, writing – original draft, writing – review and editing, investigation, methodology. **Prabhat Lamichhane:** conceptualization, supervision, writing – review and editing, writing – original draft.

## Conflicts of Interest

The authors declare no conflicts of interest.

### 1 Transparency Statement

Karan Varshney affirms that this manuscript is an honest, accurate, and transparent account of the study being reported; that no important aspects of the study have been omitted; and that any discrepancies from the study as planned (and, if relevant, registered) have been explained. All authors have read and approved the final version of the manuscript. Karan Varshney had full access to all of the data in this study and takes complete responsibility for the integrity of the data and the accuracy of the data analysis.

## Data Availability

The authors confirm that the data supporting the findings of this study are available within the article.
